# Surgery without Pain

**Published:** 1896-05

**Authors:** 


### SURGERY WITHOUT PAIN.

### IMPORTANT OPERATIONS DONE WHILE THE PATIENT IS FULLY CONSCIOUS.

A recent meeting of the Philadelphia County Medical Society was rendered particularly interesting on account of the presentation of a paper by Dr. T. Parvin on the new method of abolishing the pain of surgical operations without the necessity of employing Ether or Chloroform. This is the system suggested and practiced by the well-known German surgeon Schleich, who, by its use, has been able to perform practically all of the minor and many of the major operations of surgery without the slightest pain to the patient, and without depriving him in any other way of his consciousness.

By the method of Schleich, there are prepared three solutions of common salt, in which are dissolved different quantities of Muriate of Cocaine and Morphia. The part to be operated upon is thoroughly cleansed with an antiseptic solution, and the surface brought to a low temperature by a spray of Chloride of Ethyl. Into this area of the skin, which, by the action of the spray, has been deprived of all sensation, the salt solution containing the Cocaine and Morphine, is injected by means of a special hypodermic syringe, numerous punctures being made in all directions. This renders the deeper structures insensible to the surgeon's knife, and for a period of from twenty minutes to half an hour the patient is not conscious, so far as actual pain is concerned, of extensive cutting and sewing.

The new method differs in an important degree from the ordinary employment of hypodermic injections of Cocaine. The strength of the drug which has been used in the past is about one part in each twenty-five parts of the solution, while in the Schleich method there is often employed a strength of only one in ten thousand. In the former, however, only a few drops of the solution are employed, while in the latter the tissues surrounding the part to be operated upon are thoroughly infiltrated with the solution. With the small quantity of the Cocaine employed by Dr. Schleich, it is apparent that something more than Cocaine is responsible for the local anæsthesia so perfectly obtained. In the opinions of Drs. Keen, Ashhurst, and Morton, who discussed the merits of the new system, the infiltration of the tissues with the solution and the distention and consequent pressure upon the small nerves were responsible in a large measure for the absence of pain when the incision by the knife is made.

To indicate the manner of employing the method of Schleich, and to show the entire absence of pain, one of the surgeons had the solution inserted beneath the skin of the arm and an incision an inch long made and sewed up before the society.

In the discussion it was generally conceded, both from the results achieved by the German surgeon and the experiments made in a number of cases in this city, that a decided advance had been made in the field of anæsthetics, and that for a large number of operations the infiltration method would entirely supersede the general anæsthesia by Ether and Chloroform.—*Philadelphia Record.*

